# Dog Owners' Ideas and Strategies Regarding Dental Health in Their Dogs-Thematic Analysis of Free Text Survey Responses

**DOI:** 10.3389/fvets.2022.878162

**Published:** 2022-05-03

**Authors:** Karolina Brunius Enlund, Ann Pettersson, Ann Catrine Eldh

**Affiliations:** ^1^Department of Clinical Sciences, Swedish University of Agricultural Sciences, Uppsala, Sweden; ^2^Department of Health, Medicine and Caring Sciences, Linköping University, Uppsala, Sweden; ^3^Department of Public Health and Caring Sciences, Uppsala University, Uppsala, Sweden

**Keywords:** tooth brushing, periodontal disease, implementation, canine, dental home care

## Abstract

Periodontal disease is the most common disease in dogs over 3 years of age. In dogs, as in humans, daily tooth brushing, as a means of active dental home care, is considered the gold standard for prophylaxis and prevention of periodontal disease progression. However, the performance of adequate tooth brushing is insufficient in dogs. There is no full account as to why dog owners fail to comply with this routine, but in order to facilitate better practice, a further understanding of dog owner's perspectives is needed. The aim of this study was to investigate dog owners' ideas and strategies regarding their dogs' dental health. In a large-scale Swedish survey regarding dental health in dogs, dog owners' free text comments (*n* = 8,742) from a concluding open-ended query were analyzed using qualitative methods. Many different notions concerning dental health in dogs were identified, of which perceived importance of different diets and chewing being the most prominent. Five common themes represented dog owners' ideas and strategies regarding dental health in their dogs: what is considered to cause dental problems; what is deemed not to promote dental health; how to prevent dental problems; what impedes proper dental care, and; needs for increased knowledge and support. Contrary to existing research and knowledge in the field, the respondents commonly trusted that diet procure good dental health in the dog, as does chewing on bones. Seemingly, a range of misconceptions flourish among dog owners, indicating a need to share information and experiences, as well as support to bridge barriers to tooth brushing and other aspects that can enhance dog owners' knowledge and practice. In addition, this study highlights the need for randomized controlled trials on effects of diets and supplements on different aspects of dental health; calculus, periodontal disease, and dental fractures, including dogs of different breeds, sizes and ages. Further research is also needed with respect to which strategies that best aid dog owners, by whom the support is best provided, when, and at what time point.

## Introduction

Dental health and dental problems in dogs are a major concern, both in veterinary practice and among dog owners. From a dog owner perspective, perceived problems may include a wide range of conditions, e.g., halitosis, calculus, tooth loss or dental fractures, and owners' approaches to achieve dental health in their dog are likely multifold. However, studies are lacking on dog owners' ideas and strategies regarding dental health.

From a veterinary perspective, it is well known that a majority of dogs are affected with periodontal disease (gingivitis and periodontitis), which is initiated by dental plaque. In fact, periodontal disease is the most common disease in dogs over 3 years of age (1), with a reported prevalence ranging between 80 and 89% (2–4). If untreated, gingivitis may proceed to periodontitis, which may eventually result in tooth loss. Dogs of smaller size and of certain breeds are predisposed to periodontal disease, and the prevalence also increases with age (2, 5–11). However, contrary to humans, caries (tooth decay) is rare in dogs (6). Another issue is that dental plaque, if left undisturbed, mineralizes into dental calculus (tartar) (12). Calculus is not pathological in itself but facilitates the adhesion of dental plaque, thus increasing the risk of periodontal disease. However, many dog owners lack knowledge concerning the distinction between plaque, calculus, gingivitis, and periodontitis, and associated signs.

Among dog owners, the practice of giving the dog a variety of bones (natural chews, e.g., rawhide, or natural skeletal details) to chew is very common, both as a means of dental care and as a way of pastime (13). Yet, the evidence of chewing of bones being beneficial for dental health is very limited (14–16). Rather, in dogs, as in humans, daily tooth brushing as a means of active dental home care is considered the gold standard for prophylaxis and prevention of periodontal disease progression (17–21). However, the performance of adequate tooth brushing is insufficient in dogs (22, 23).

In a large-scale quantitative Swedish survey on dental care in dogs, we have shown that only 4% of Swedish dog owners brushed their dogs' teeth daily (22, 24). So far, to the authors' knowledge, no other studies have attended to owners' beliefs and attitudes to dental care for dogs from a larger perspective. With little known regarding the barriers for adequate dental home care, there are limited opportunities to facilitate further implementation of better practice; rather, at this point, it is vital to better understand the dog owner perspective (25). With such comprehension, we can move beyond standard knowledge dissemination and tailor implementation strategies where needed, i.e., in the home environment (26, 27).

The aim of this study was to investigate dog owners' ideas and strategies regarding dental health in their dogs.

## Materials and Methods

This is a study of qualitative design, originating from a large-scale Swedish survey regarding dental health in dogs, conducted in April 2017 (22, 24, 28). The survey comprised a final open-ended query with opportunity for free text comments previously not addressed; for this study, the 8,742 accounts were analyzed using qualitative analysis.

The use of validated questionnaire surveys is a well-established method for investigating attitudes and practices (28–30). Open-ended questions asking for additional comments are common in surveys, and give respondents an opportunity to provide additional information, elaborate responses to closed questions and identify new issues not captured in the closed questions (31, 32). While this information is not always analyzed or presented by researchers, it can be a valuable data source (31).

### Data Collection

Construction and validation of the survey has been described in detail previously (28). In brief, a nationwide questionnaire survey to dog owners was conducted with structured questions regarding dental health and dental home care in their dog. The survey included, in addition to background questions on dog and owner, items on attitudes to and use of: active dental home care, i.e., tooth brushing; passive dental home care, including e.g., so-called dental food, manufactured dental chews, and natural chewing bones; appraisal of the dogs dental health, and; veterinary information and interventions for the dogs dental health (22, 24, 33). The details of the development and validation, including non-response analysis, of the questionnaire is described previously (28), and also the results of the closed questions (22, 24, 33). The questionnaire consisted of 54 to 68 questions, number depending on previous answers.

At the end of the survey, the questionnaire displayed an open-ended question phrased as: “If you have anything additional that you want to share, please feel free to do so here”. The invitation yielded 8,742 unique responses, which were considered for this study; however, free text responses regarding other issues than dental health care were excluded prior to the analysis, such as records of the dog having, not having, or having had dental problems, or an appreciation of the survey (in general or a particular topic).

### Setting and Sample

The survey invitation was sent to all dog owners with an email address registered with the Swedish Board of Agriculture or the Swedish Kennel Club (*n* = 209,263). The questionnaire was distributed via the web platform Netigate (Netigate AB, Stockholm, Sweden). The respondents were unidentified during and after the data collection and analysis, and the questionnaire could only be answered once per link. The overall response rate was 32% (*n* = 66,434), of which 13% (*n* = 8,742) gave a free text comment in the final open question.

Dog owners were 50 ± 13 years of age. The majority (75%) were women, and almost half (46%) of all dog owners lived in urban counties (Stockholm, Skåne, Västra Götaland). More than two-thirds (70%) were employed or self-employed. Half (49%) had studied at a university, and almost one in four (23%) reported that they worked within a healthcare profession. Moreover, one in 12 (8%) was a dog breeder. The dogs were 5 ± 4 years of age (mean ± SD). All breed groups were represented. One-third (33%) of dogs weighed <10 kg and the majority (78%) of all dogs were intact (22).

### Data Analysis

The free text responses were inserted verbatim in Microsoft Excel prior to analysis, with each response yielding a separate article. An inductive, data-driven approach was applied to the free text answers, corresponding to the study aim, inspired by thematic analysis in the following process and phases (34):

1. Data familiarization, including a reading of a random 500 free text responses to become familiar with the data set.
2. A subsequent detailed repeated reading of a random selection of 200 free text responses to identify common content. Multiple cycles of consecutive coding generated a semifinal set of 33 codes.
3. These codes were then applied to another random 500 free text responses in a critical assessment of the codes. This phase rendered 7 more codes, i.e., 40 codes for a reliable coding matrix.
4. The full set of 40 codes were then applied for the entire set of written comments. When experiences represented more than one perspective, the thematic analysis endorsed respondents' comments to be reclaimed; that is, if certain issues were raised as both enabling and hindering dental health, such meaning units could be incorporated in two subthemes.
5. The codes and corresponding content were formed into 18 subthemes.
6. Subsequently, the 18 subthemes were formed into five themes.
7. Quotations were selected to illustrate the dog owners' shared experiences (35), the number reported in parenthesis after each quotation to illustrate the spread across the data set.

Step 1, 3, 4, and 7 were performed by author KBE, in dialogue with author ACE. Steps 2, 5, 6, and 8 were performed by authors KBE and ACE, with frequent discourse as for the best understanding of the data and the emerging findings (34).

## Results

Five common themes were established by means of altogether 18 subthemes (ranging from two to seven for each theme) to represent the dog owners' ideas and strategies. An overall view of the subthemes and themes is provided in Table 1. The more detailed descriptions of the content of each theme is further explicated below, subthemes underlined.

**Table 1:** Themes and subthemes of the thematic analysis.

***Theme***	***Subtheme***
Dog owners' ideas of what is causing dental problems	Eating the wrong food
	Chewing marrowbones
	Aging
	Dog having poor genetic traits, being of small size, specific breed or family
Dog owners' ideas of what is not promoting dental health	Tooth brushing
	Using dental products
Dog owners' ideas and strategies for preventing dental problems	Eating the right food
	Chewing
	Using dietary supplements or dental products
	Using dental scaler at home or at the veterinary clinic
	Checking dental status regularly
	Tooth brushing
	Dog having favorable genetic traits, being a large size, specific breed or family
Dog owners' ideas of what is impeding proper dental care	Difficulties with tooth brushing
	Dog doesn't like /tolerate bones/chews
	Dental care costs
Dog owners' proposed needs for increased knowledge and support	More and better information
	Better dental products

### Dog Owners' Ideas of What Is Causing Dental Problems

A large number of dog owners perceived that a healthy/good diet is imperative for good dental health, and conversely that a bad/wrong diet causes dental problems. However, what was considered a bad diet differs vastly and was not always specified in the present study.

Dry kibble diets in general, or specific brands, or in specific feeding regimes, e.g., if given moistened, were by some considered detrimental for dental health.

A multitude of owners strongly advised against providing dogs with marrowbones due to the risk of dental fractures, trauma and teeth wear and reported personal experiences from veterinary consultations and surgical procedures However, other dog owners considered marrowbones beneficial for dental health.

”*Marrow bones are too hard and can cause tooth damage, however, I often give RAW ribs and chicken wings for dental health [….].” (No 217)*

Old age was considered a risk factor for dental disease, and dog owners could specify e.g. that their dog had good dental health despite old age, or that it is normal for dogs to have more dental calculus when aging.

”*The vet thinks my dog has good teeth for his age” (No 4550)*

In the free text comments, many dog owners gave specified information about the dog's dental status. E.g., many commented that their dog was only a pup or a young dog and therefore did not have any dental problems and consequently were not in need of dental care. This opinion is, however, unfortunate since prevention of dental disease is better than cure (36).

As mentioned, heredity and breed were considered very important for dogs' dental health. Some dog owners reported noticing a substantial difference between dental health in different breeds. On the other hand, dental health was also reported to differ between individuals within the same breed. Sometimes the inclination for build-up of dental calculus, tooth loss, gum disease or periodontitis was specifically mentioned, but often specific aspects of dental health was not specified further in relation to breed. “Small breeds” were commonly described as having more dental problems than “large breeds”, but also specific breeds, e.g., Chihuahua, Yorkshire terrier, or just “my breed” have/have not a predisposition for dental problems.

”*My experience regarding dental health in my breed is that it is hereditary and that we have major problems in the breed regarding dental health” (No 6894)*

Malocclusions such as overbite, underbite or crowded teeth, or other anatomical variations such as a small mouth, were sometimes mentioned as predisposing for dental problems.

### Dog Owners' Ideas of What Is Not Promoting Dental Health

As previously mentioned, tooth brushing in dogs was covered in the closed questions of the questionnaire. However, many dog owners elaborated on this practice in the open-ended free text question at the end of the survey. The attitude toward tooth brushing was mainly positive (22). However, many dog owners considered tooth brushing in dogs to be unnecessary or even unnatural.

Dog owners commonly remarked that they would of course brush their dogs' teeth if it was needed, which currently it was not since the dental status was excellent.

“*I have never had to brush the teeth of any of my dogs but would of course do so if there was a problem.” (No 5773)*

Some considered tooth brushing unnatural and would never brush their dog's teeth, and some commented that it is never needed as long as the dog is of a healthy breed and is given a natural diet.

“*If a dog needs daily tooth brushing, there is something wrong with food or breeding. Wolves do not brush their teeth...” (No 4398)*

A few commented that tooth brushing was ineffective, they had tried and brushed every day but the dog still had dental problems or kept loosing teeth. Other remarks concerned the uselessness of dental products or chews of a specific brand. Some dog owners were of the opinion that the product had no beneficial effects on dental health, some that the product was ineffective because their dog ate it too fast and didn't chew sufficiently on it, and some that they were bad for their dog's general health. However, some dog owners also remarked that the product improved dental health.

### Dog Owners' Ideas and Strategies for Preventing Dental Problems

The most common subtheme among the free text answers was the importance of diet and chewing for dental health in dogs. Several respondents expressed dissatisfaction with the fact that the questionnaire did not ask about feeding habits. “A healthy diet” was considered by numerous dog owners as imperative for good dental health. However, what characterizes a “healthy diet”, was not always clear or specified further.

Two main reasons for the perceived dental benefit of certain diets were discovered in the analysis. Some considered natural chews (such as rawhide or pigs ears or antlers) or raw meaty bones to be effective to improve dental health, through the actual chewing. On the other hand, some considered specific diets to be beneficial for their dog's dental health without the mechanical action of chewing (such as a sugar free diet, or a diet free from carbohydrates or cereals or only home cooked food, or a diet free from human food/left-overs, or no eating between meals). Standard dry kibble was perceived by some as being good for dental health by being hard in texture, by others by having a healthy composition (whereas other dog owners, as mentioned, considered kibble to be unhealthy).

”*My dogs are never allowed to eat anything with sugar in it” (No 438)*

One common code within the subtheme “Diet” was the importance of raw meat-based diets (RMBDs), primarily BARF (Biologically Appropriate Raw Food / Bones and Raw Food), and/or raw meaty bones to promote dental health in dogs. Many commented on decreased halitosis or decrease in dental calculus since they started with this diet.

”*After the dog started with the BARF-diet, tartar and bad breath are completely gone!” (No 2474)*

The type of raw meaty bones was frequently specified, e.g., ribs, neck vertebrae and chicken necks, and often combined with the conception of marrowbones as being distinctly bad for the dog's teeth because of the hardness and shape, with risk of dental fractures and entrapped jaw. However, others reported using raw meaty bones in conjunction with using marrowbones.

“*My dogs have clean healthy teeth due to free access to rawhide chewing bones.” (No 6617)*

Dog owners commented frequently on the benefits of chewing, either in general that the chewing in itself was beneficial, or in particular on specified items. Marrowbones, or just “bones” (which may be anything from smoke-dried marrowbones, to raw meaty bones, to chicken necks), natural chews (such as rawhide chews, pig ears), antlers from moose/ reindeer/ deer, hooves etc., and also brand-named dental chews as well as carrots and hard biscuits/crispbread were all stated as beneficial for dental health.

”*My dog eats raw carrots every day. I think that's why she has good teeth. We usually get praise for her fine teeth at the vet.”(No 6407)*

In addition, chewing on wooden sticks, branches, logs of wood etc. was considered by many to be very beneficial for dental health in dogs. There were also occasional reports on other chewing items, e.g., tires, ropes, textile of fleece or wool, basketballs, training bite suits and nylon bones.

”*For me, it is important that the dog can play with wooden sticks and pull branches, it's a good way to clean teeth in a natural way.” (No 2157)*

Dietary supplements that were commonly used for improving dental health was e.g., algae products and enzymatic toothpastes, but also other tooth pastes/gels and water additives. Sometimes just “powder” or “feed supplement” was reported. In addition, coconut oil, salt water or aloe vera was used by some respondents when cleaning their pet's teeth. Also, there were sporadic reports of using enzymatic chews, green-lipped mussel, colloidal silver, essential oils and magnetic plate for dental health.

Regular checkups at the veterinary clinic was often mentioned in the comments and was considered important to discover problems. Many dog owners also specified in comments that the dog was in excellent dental health according to their veterinarian. Also, regular controls at home of dental status was deemed important for good dental health, as well as training in handling early in the dog's life to be able to inspect and care for the dog's teeth. Several dog owners also reported that professional dental cleanings were performed on their dog at the veterinary clinic, when problems such as calculus or periodontitis were apparent, or in conjunction with anesthesia for other, unrelated, medical issues.

“*The teeth are checked regularly by me and we go to the vet when / if I see that tartar is starting to appear.” (No 282)*

A dental (hand) scaler used at home was a common way to remove calculus and perceived as caring for the dog's teeth. This could be used regularly, or on an as-needed basis. Sometimes substances claiming to soften calculus were also specified as facilitating removal of calculus with the dental scaler.

”*I have always used a tartar scaler on my older dogs when they got some tartar.” (No 592)*

In addition to being reported in the closed questions (22), tooth brushing could be suggested as a natural way of caring for the dog's teeth. Dog owners e.g., described their routine regarding tooth brushing, e.g., using an electric tooth brush. Some specified that they brushed when needed, e.g., if the dog had eaten “yucky stuff”.

Having a healthy breed of dog or having a large dog was considered as preventing dental problems. However, many dog owners perceived that there was also an individual difference between dogs of the same breed or in the same household.

”*Overall, what I have seen of the dogs of this breed, I reckon they have good dental health, but on an individual basis some get tartar more easily” (No 6380)*

### Dog Owners' Ideas of What Is Impeding Proper Dental Care

Difficulties to perform tooth brushing due to an unwilling or scared dog was noted by several dog owners in the free text. Some dogs were even aggressive. Dog owners could comment that they had not started training the dog to accept tooth brushing as a pup, and that it was now too late to start. Some owners had also acquired their dog as a grown dog, stating this as the reason for problems with dental health or dental care. Other reasons mentioned were breed (aggressive, small, brachycephalic), technical difficulties and difficulties with incorporating the routine, implementing the practice and motivation.

Another obstacle for providing dental care, according to several dog owners, was that the dog could or would not chew on specific items, such as marrowbones, bones, dental chews etc. The reasons could be allergies, or that the dog had a sensitive stomach requiring specific veterinary diets, or that the dog was prone to gastrointestinal problems in response to different edible dental products. In addition, some dog owners stated that the dog was not interested in chewing, no matter what they tried, which was considered a problem for dental health.

”* The dog is allergic to wheat. Doesn't tolerate chewing bones so it is difficult then to give him something to chew on for the sake of the teeth.” (No 3403)*

High costs for dental cleaning at the veterinary clinic was a reoccurring subtheme under obstacles. According to some respondents, if the cost was lower, dog owners would more often bring their dog to the veterinary clinic for professional dental cleaning, and therefore lower costs would enable more dogs to obtain help. In addition, the fact that pet insurance companies commonly do not cover dental costs was considered by some as a big problem.

”* Dental hygiene should be included in the dog insurance, then more people would visit the vet more often.” (No 2121)*

### Dog Owners' Proposed Needs for Increased Knowledge and Support

Another emerging theme was the need for more information regarding dental care and dental health in dogs. This lack of information was mostly commented on in general terms, but sometimes also in detail, for instance that breeders and veterinarians in particular should inform and educate dog owners regarding dental care. Available information was also sometimes conceived as biased, e.g., that commercial interests were trying to influence dog owners, or that different sources provided different advice. There was a perception/sense of not knowing whom to trust.

”*Would like to take a tooth brushing course;)! Have a bad conscience.” (No 2955)*

Besides the need for reliable advice, some owners expressed a need for better products, e.g., smaller tooth brushes, dental chews with higher palatability, products for dental health that are really efficient when tooth brushing is not possible.

## Discussion

As illustrated by the sheer amount of free text answers in our study, >8,000, there is a genuine concern and great engagement in their dog's dental (as well as general) health and well-being among dog owners. The richness and depth of data from open-ended survey questions is more limited than data obtained from interviews or focus groups (37). It does, however, incorporate a greater breadth of experience from a wider range of respondents. Although the findings do not represent a full account of all dog owners perspectives yet provides a rare outlook on what many dog owners suppose and thus may act on in daily dental care of their dogs. As such, they broaden the perspectives of the quantitative findings, of closed survey questions (22, 24), yielding prospects for further research and practice development.

The respondents who chose to answer the open question may not be representative of the total sample population. They may e.g., have a greater interest in the topic of the survey. This should be taken into account when interpreting the results (31). Also, the study was performed in a Swedish social and cultural context which should be taken into account in any international comparisons. However, by incorporating the full data set in the analysis, completed in alignment with a well-known method and in recurrent discussions between experts in the area investigated and in methodology, trustworthiness was sought (38).

### Dental Health and Dental Problems

It is well-established that there is a high prevalence of periodontal disease, dental fractures and malocclusions in dogs (39), conditions that are known to be detrimental to dental health. However, what was apparent in the present study was that dental health may mean different things to dog owners: many owners mentioned halitosis and dental calculus as examples of dental problems, but also gum disease (periodontitis), and previous traumatic injuries resulting in fractured teeth and visits to the veterinary clinic. However, dental calculus is not pathological in itself, a fact that may not be known among the general public. Even so, calculus is often, but not always, seen in conjunction with periodontal disease (12). In addition, heavy dental calculus is a sign of a lack of dental home care, even though the disposition/tendency for build-up of dental calculus differs between individuals (40). It is likely that dog owners experiencing calculus without periodontitis or tooth loss in their dog consider dental care of less importance than owners with previous experience of tooth loss in their dog do.

Moreover, halitosis (bad breath) is a symptom and not a disease in itself. However, the most common cause of halitosis is periodontal disease (12). Besides being a possible symptom of disease, it could negatively affect the social interaction in the companion animal–owner relationship (41). Therefore, halitosis should not be ignored but treated according to cause.

### Dental (Home) Care

#### Feed and Chew

A major finding in the present study was dog owners' views on feed and bones as extremely important for dental health. Interestingly, despite being the center of much attention in the commercial feed industry, there is a scarcity of studies on the importance of different diets or individual chewing habits, for dental health (including calculus and periodontal disease) (33).

In humans, caries (tooth decay) is the most common dental disease and much advice within human dentistry focuses on prophylactic measures against this disease. However, dogs very rarely get caries (6), and avoiding sugar, carbs or eating between meals likely represent a misconception, directly transmitted from human dentistry. In fact, to the authors' knowledge, no evidence exist that such measures could prevent periodontal disease in dogs.

Dry kibble is presently the most common basis for feeding dogs in Sweden, but research is contradictory regarding if standard dry kibble is beneficial or not for dental health (14, 42).

Although so-called “dental foods” with a specifically designed texture to mechanically remove plaque as the dog chews, has been shown to decrease plaque and calculus (43), standard, dry, pelleted feed usually crumbles when chewed and is therefore not likely to be effective on dental plaque, contrary to many dog owners' ideas. Other dog owners express that dry kibble is bad for the dogs' dental and/or general health, illustrating the diversity in dog owners' strategies regarding dental health.

A multitude of respondents voiced a “natural” diet, or meat and bones, as imperative for dental health. In line with this, other dog owners stated that their dog suffered from poor dental health because they didn't like to chew. Chewing bones as a way of mechanical cleansing of teeth was mentioned by many dog owners, others proposed that chewing in itself was beneficial for dental health and sometimes dog owner's didn't specify the reason why this food was perceived as beneficial.

Both commercial and home-prepared raw meat-based diets (RMBDs) are becoming increasingly popular, and are often claimed to have superior nutritional quality and significant health benefits by its advocates. Anecdotal benefits for RMBDs according to Freeman et al. include “better palatability of these diets, cleaner teeth from chewing bones as a part of these diets, a shiny coat, and owner perception that they are providing their pet with a more natural diet” (44). However, there is a scarcity of published studies and claims of benefits are largely based on anecdotal rather than scientific evidence (45, 46). On the contrary, one study showed no improved dental health in dogs fed a “natural diet” (47). The perception of meat being the only natural diet for dogs as it is for their forefather the wolf, may be questioned since dogs differ from wolves specifically in their metabolism of starch (48).

Some advocates of RMBDs provide their dogs with marrowbones together with raw meat and non-weight bearing skeletal details, whereas other dog owners that feed RMBDs commented that marrowbones may cause dental fractures. This is common knowledge in the veterinary community and a reason why veterinarians often advise against marrowbones (39). In fact, traumatic dental fractures are very common, with a reported prevalence of up to 26% (49). This may often be caused by chewing on hard objects, such as marrowbones. Other dental risks with feeding some hard skeletal details is impaction in the oral cavity and subsequent localized periodontal disease, or entrapped jaw by circular marrow bone.

Contradictory, many dog owners used marrowbones as a way of caring for their dogs teeth. Evidently, not all dogs that chew on marrowbones suffer from dental fractures. The role of chewing in development of dental fractures is still controversial, due to the lack of research in the area, and likely depends on several things, e.g., crown height to diameter ratio (50). It may also be hypothesized that size of the chewing bones, individual occlusion, chewing intensity and individual chewing patterns also play a role in the development of dental fractures. Interestingly, some studies have indicated that the use of bones can decrease dental calculus (15, 51). These studies have, however, been small and more evidence is needed.

Worth noticing is the difference between calculus, plaque and periodontal disease: even if chewing bones may help control calculus in some dogs, there is a noticeable lack of evidence regarding the use of chewing bones for prevention of periodontitis. Given the absence of evidence, veterinarians and veterinary nurses should not be recommending RMBD or chewing of bones to support periodontal health until more evidence is generated (52).

The use of carrots for promoting dental health was common in the present study, and interestingly also recommended by veterinarians according to some owners. This practice is not evidence-based, in fact studies in humans have shown that neither the chewing of carrots nor apples have any positive effect on gingival health (53, 54).

Our results also showed that chewing on wooden sticks, branches, logs of wood etc. was suggested to be beneficial for dental health in dogs. To our knowledge, there are hitherto no studies on the effect of chewing wooden sticks on dental health in dogs, although there may be a risk of wood splinters between teeth and trauma to the oral cavity. It could, however, also be hypothesized that the effect may be comparable to that of some abrasive materials on the buccal side of premolars/molars, depending on chewing pattern.

Some commercial dental products have shown a limited effect on plaque and/or calculus in scientific studies (43). However, the majority of marketed products have not been properly evaluated in peer-reviewed publications and their use is thereby not evidence-based. A dental chew of a specific named brand was by some dog owners in the study conceived as bad for the dog's health, by causing digestive trouble (vomiting, diarrhea), skin problems, obesity or just being unhealthy and sugary. This was mentioned as a reason for not giving it, although some of these dog owners thought it might be beneficial for the dog's dental health. Some dog owners also specified that they perceived it as helpful for the dog's dental health, and some dog owners stated that they used it without really believing it worked. On the other hand, other dog owners disregarded the product as useless, illustrating the diversity of opinions regarding what is actually beneficial for dental health.

#### Breed/Heredity, Size and Age

Being of small breed and having a small body size was expressed by many as predisposing to poor dental health, in line with previous and current research (2, 5, 11, 24). Experiences of large individual differences within the same breed, in the same family, and/or under the same living conditions were also expressed. However, in the quantitative part of the questionnaire, as many as 27% did not know or stated breed/heredity of minor importance, showing a knowledge gap regarding heredity of some dental diseases (24). Old age was mentioned as a “natural” cause of poor dental health. While it is true that periodontal disease prevalence increases with age, brushing the dogs teeth can often prevent this deterioration in dental health (39).

#### Checkups

Regular checkups was mentioned by many respondents as important to detect dental problems early. International guidelines state that regular professional examinations are recommended to detect and treat problems, including malocclusions and dental fractures (39). Periodontal disease, on the other hand, cannot be prevented by regular checkups. Instead, dental home care is required several times per week (17, 39). In addition, it is better to prevent periodontal disease than to treat already existing disease, in line with human dentistry practice where a combination of daily tooth brushing and regular professional dental care is the basis for good oral health.

#### Brushing

Even if the quantitative report on attitudes and practices regarding tooth brushing has been thoroughly discussed previously (22), many dog owners elaborated further on this in free text.

Several dog owners stated that brushing was unnecessary, since the dog had good, shiny or white teeth. However, many also stated that they would of course brush their dog's teeth, or treat the dog, if it showed signs of any problems. This attitude is however unfortunate, since prevention is usually a more efficient strategy than trying to treat already manifest disease. Other reasons for not brushing were that the breed was a healthy one without inherited dental problems, that they had many dogs or working/hunting dogs or that they had never had any problems in the past. The opposite was also stated, that the owner had had dogs in the past with problems, and therefore was more dedicated to dental care.

The perception of tooth brushing in dogs as unnatural was also expressed, and that wild animals don't brush their teeth. While this is true, dental problems is also very common in wild animals, including wolfs and wild dogs (55–57). It may also be worth noticing that tooth brushing is a relatively new habit in human dentistry, with the first nylon brushes introduced only in the 1930s, illustrating that a perceived unnaturalness does not exclude usefulness.

Difficulties with brushing routines were elaborated on by several dog owners. In the questionnaire's quantitative data, we found that 25 % of the dog owners had difficulties inspecting their dogs teeth, most commonly because of an unwilling dog or that it was technically difficult (24). Among the free-text answers, reported tooth brushing difficulties included that the dog had been adopted as an adult, or that it had not been trained as pup. While it may in fact be difficult to train a scared or unaccustomed dog, it is by no means impossible, which may be worth informing dog owners about (58). We hypothesize that tooth brushing of dogs may become a more natural part of daily care in the future, similar to regular grooming, bathing and nail clipping.

Daily tooth brushing remains the evidence-based gold standard for dental home care, whereas passive dental home care, such as dental chews, may be used as a complement, not replacement (39). More studies in the future about risk individuals (breed, size) may make it possible to target individuals at risk to receive extra information and support.

#### Dental Care Costs and Need for Knowledge Implementation

The cost of dental care was mentioned by a multitude of dog owners as a hindrance for good dental care. Both veterinary costs and the fact that dental cleanings are not covered by the insurance were mentioned. In Sweden, veterinary dental care may be partly substantiated if the dog owner has full insurance for the dog but even so, dental calculus and periodontal disease are not covered but fully paid by the dog owner. However, a fact that many dog owners may be unaware of, is that professional dental cleanings without subsequent dental home care mainly serve a cosmetic purpose.

In order to improve dental health among dogs, we propose that dog owners need further support. In fact, the insights provided in our survey suggest that there are several issues that need further attention: misconceptions, a lack of dental health literacy, and deficiencies in dental care routines.

Rather, some dog owners request more, and more unbiased, information on dental care. We propose that breed clubs as well as veterinary clinics may include more dental care information in their material to breeders as well as to new dog owners. It would indubitably be helpful for owners of breeds known to be predisposed to periodontal disease to receive more targeted information. We also propose that veterinary nurses could play a more active role in educating and discussing dental home care with dog owners, similar to human dentistry where dental hygienists regularly examine, follow up and discuss dental home care.

To date, the most common approach to improve dental health is by dissemination information, through veterinary health professionals, web-pages, books or journals (22). However, a lack of knowledge is likely only a portion of what constitutes barriers to improved dog dental health. Rather, to facilitate better practice, the misconceptions, e.g., about importance of different diets, need attention, which is a delicate matter. A change in attitudes is a vital step in the process of changing behaviors: in this case adopting more frequent tooth brushing of the dog (59). This requires a recognition of the dog owners' ambition to care for the dog, in combination with a (preferably tailored) strategy to master both why and how to proceed. The latter includes knowing how to perform tooth brushing, and ways to work around when the dog is unwilling to have the teeth brushed. Effectively aiding dog owners in adopting a functioning, persistent routine should preferably be a joint venture, engaging dog owners and breeders, veterinarians and veterinary nurses, dog groomers, course organizers, and publishers of dog journals etc. Further research is also needed with respect to which strategies aid dog owners best, by whom the support is best provided, when, and at what time point (27).

## Conclusions

Contrary to contemporary research and knowledge in the field, the dog owners responding to our survey commonly trusted that certain diets procure good dental health in the dog, as does chewing on bones. This indicates a need for opportunities to share information and experiences, as well as support to bridge barriers to tooth brushing and other aspects that can enhance dog owners' knowledge and practice. In addition, this study emphasizes the lack of evidence regarding many products and procedures advocated for improving dental health in dogs and highlights the need for high-quality intervention studies, preferably in long-term randomized controlled trials including dogs of different breeds, sizes and ages, to investigate effect of diet and supplements on different aspects (calculus, periodontal disease, fractures) of dental health.

## Data Availability Statement

The datasets presented in this article are not readily available because the comments may include statements that make them identifiable, therefore they are strictly confidential according to national legislation. Requests to access the datasets should be directed to karolina.enlund@slu.se.

## Ethics Statement

The studies involving human participants were reviewed and approved by the Regional Ethical Review Board in Uppsala (Dnr 2017/035). Written informed consent for participation was not required for this study in accordance with the national legislation and the institutional requirements.

## Author Contributions

KE and AP: conception and design of study. AP: funding. KE: coding of data and writing original draft. KE and AE: forming of subthemes, themes, and analysis. All authors contributed to manuscript revision, read, and approved the submitted version.

## Funding

This work was funded by the Greater Stockholm Veterinary Hospital Foundation and the Swedish Association for the Protection of Animals to AP. The funders had no role in study design, data collection and analysis, decision to publish, or preparation of the manuscript.

## Conflict of Interest

The authors declare that the research was conducted in the absence of any commercial or financial relationships that could be construed as a potential conflict of interest.

## Publisher's Note

All claims expressed in this article are solely those of the authors and do not necessarily represent those of their affiliated organizations, or those of the publisher, the editors and the reviewers. Any product that may be evaluated in this article, or claim that may be made by its manufacturer, is not guaranteed or endorsed by the publisher.

## References

[1] Niemiec B. Veterinary Periodontology. Ames: John Wiley & Sons (2012). 10.1002/9781118705018

[2] Hamp SE Olsson SE Farsomadsen K Viklands P Fornell J. A macroscopic and radiologic investigation of dental diseases of the dog. Vet Radiol. (1984) 25:86–92. 10.1111/j.1740-8261.1984.tb01916.x

[3] Kortegaard HE Eriksen T Baelum V. Periodontal disease in research beagle dogs–an epidemiological study. J Small Anim Pract. (2008) 49:610–6. 18793256 10.1111/j.1748-5827.2008.00609.x

[4] Fernandes NA Borges APB Reis ECC Sepúlveda RV Pontes KCdS. Prevalence of periodontal disease in dogs and owners' level of awareness - a prospective clinical trial. Revista Ceres. (2012) 59:446–51. 10.1590/S0034-737X2012000400003

[5] Harvey CE Shofer FS Laster L. Association of age and body weight with periodontal disease in North American dogs. J Vet Dent. (1994) 11:94–105. 10.1177/089875649401100301 9693607

[6] Kyllar M Witter K. Prevalence of dental disorders in pet dogs. Vet Med. (2005) 50:496–505. 10.17221/5654-VETMED

[7] Hoffmann T Gaengler P. Epidemiology of periodontal disease in poodles. J Small Anim Pract. (1996) 37:309–16. 10.1111/j.1748-5827.1996.tb02396.x 8840250

[8] Marshall MD Wallis CV Milella L Colyer A Tweedie AD Harris S. longitudinal assessment of periodontal disease in 52 miniature schnauzers. BMC Vet Res. (2014) 10:166. 10.1186/1746-6148-10-166 25179569 PMC4236762

[9] O'Neill DG Rooney NJ Brock C Church DB Brodbelt DC Pegram C. Greyhounds under general veterinary care in the UK during 2016: demography and common disorders. Canine Genet Epidemiol. (2019) 6:4. 10.1186/s40575-019-0072-5 31179010 PMC6547581

[10] Wallis C Pesci I Colyer A Milella L Southerden P Holcombe LJ. A longitudinal assessment of periodontal disease in Yorkshire terriers. BMC Vet Res. (2019) 15:207. 10.1186/s12917-019-1923-8 31226991 PMC6588847

[11] Wallis C Holcombe L. A review of the frequency and impact of periodontal disease in dogs. J Small Anim Pract. (2020) 61:529–40. 10.1111/jsap.13218 32955734

[12] Reiter AM Gracis M. BSAVA Manual of Canine and Feline Dentistry and Oral Surgery: British Small Animal Veterinary Association. Gloucester: BSAVA (2018) 392. 10.22233/20412495.1018.24

[13] Morelli G Marchesini G Contiero B Fusi E Diez M Ricci R. Survey of dog owners' attitudes toward treats. J Appl Anim Welf Sci. (2020) 23:1–9. 10.1080/10888705.2019.1579095 30773055

[14] Harvey CE Shofer FS Laster L. Correlation of diet, other chewing activities and periodontal disease in North American client-owned dogs. J Vet Dent. (1996) 13:101–5. 10.1177/089875649601300304 9520786

[15] Marx FR Machado GS Pezzali JG Marcolla CS Kessler AM Ahlstrom O. Raw beef bones as chewing items to reduce dental calculus in Beagle dogs. Aust Vet J. (2016) 94:18–23. 10.1111/avj.12394 26814157

[16] Logan EI. Dietary influences on periodontal health in dogs and cats. Vet Clin North Am Small Anim Pract. (2006) 36:1385–401. 10.1016/j.cvsm.2006.09.002 17085242

[17] Harvey C Serfilippi L Barnvos D. Effect of frequency of brushing teeth on plaque and calculus accumulation, and gingivitis in dogs. J Vet Dent. (2015) 32:16–21. 10.1177/089875641503200102 26197686

[18] Tromp JAH Jansen J Pilot T. Gingival health and frequency of tooth brushing in the beagle dog model. J Clin Periodontol. (1986) 13:164–8. 10.1111/j.1600-051X.1986.tb01451.x 3455949

[19] Tromp JAH van Rijn LJ Jansen J. Experimental gingivitis and frequency of tooth brushing in the beagle dog model. J Clin Periodontol. (1986) 13:190–4. 10.1111/j.1600-051X.1986.tb01458.x 3457807

[20] Gorrel C. Home care: products and techniques. Clin Tech Small Anim Pract. (2000) 15:226–31. 10.1053/svms.2000.21625 11269998

[21] Gorrel C Rawlings JM. The role of tooth-brushing and diet in the maintenance of periodontal health in dogs. J Vet Dent. (1996) 13:139–43. 10.1177/089875649601300405 9520789

[22] Enlund KB Brunius C Hanson J Hagman R Höglund OV Gustås P. Dental home care in dogs-a questionnaire study among Swedish dog owners, veterinarians and veterinary nurses. BMC Vet Res. (2020) 16:90. 10.1186/s12917-020-02281-y 32188446 PMC7081671

[23] Ipsos. Most (95%) Pet Owners Brush Their Own Teeth Daily, But Few Brush Their Dog's (8%) or Cat's (4%) Teeth on a Daily Basis (2016). Available online at: https://www.ipsos.com/en-ca/news-polls/most-95-pet-owners-brush-their-own-teeth-daily-few-brush-their-dogs-8-or-cats-4-teeth-daily-basis (accessed February 26, 2019).

[24] Enlund KB Brunius C Hanson J Hagman R Höglund OV Gustås P. Dog owners' perspectives on canine dental health—a questionnaire study in Sweden. Front Vet Sci. (2020) 7:298. 10.3389/fvets.2020.00298 32582779 PMC7297050

[25] Nilsen P Birken SA. Handbook on Implementation Science. Cheltenham: Edward Elgar Publishing (2020). 10.4337/9781788975995

[26] Wensing M. Implementation science in healthcare: Introduction and perspective. Zeitschrift für Evidenz, Fortbildung und Qualität im Gesundheitswesen. (2015) 109:97–102. 10.1016/j.zefq.2015.02.014 26028446

[27] Skivington K Matthews L Simpson SA Craig P Baird J Blazeby JM. Framework for the development and evaluation of complex interventions: gap analysis, workshop and consultation-informed update. Health Technol Assess. (2021) 25:1–132. 10.3310/hta25570 34590577 PMC7614019

[28] Brunius Enlund K Brunius C Hanson J Hagman R Hoglund OV Gustas P. Development and validation of two questionnaires: Dental home care and dental health in Swedish dogs. PLoS ONE. (2019) 14:e0204581. 10.1371/journal.pone.0204581 30682017 PMC6347148

[29] Marsden PV Wright JD. Handbook of Survey Research. Bingley: Emerald (2010).

[30] Tourangeau R Rips LJ Rasinski K. The Psychology of Survey Response. Cambridge: Cambridge University Press (2000). 10.1017/CBO9780511819322

[31] Rich JL Chojenta C Loxton D. Quality, rigour and usefulness of free-text comments collected by a large population based longitudinal study-ALSWH. PLoS ONE. (2013) 8:e68832. 10.1371/journal.pone.0068832 23874784 PMC3708890

[32] O'Cathain A Thomas KJ. “Any other comments?” open questions on questionnaires–a bane or a bonus to research? BMC Med Res Methodol. (2004) 4:25. 10.1186/1471-2288-4-25 15533249 PMC533875

[33] Enlund KB. Dental care in dogs: a survey of Swedish dog owners, veterinarians and veterinary nurses. Doctoral thesis. Acta Universitatis Agriculturae Sueciae. (2021).

[34] Braun V Clarke V. Using thematic analysis in psychology. Qual Res Psychol. (2006) 3:77–101. 10.1191/1478088706qp063oa

[35] Eldh AC Årestedt L Berterö C. Quotations in qualitative studies: reflections on constituents, custom, and purpose. Int J Qual Methods. (2020) 19:1609406920969268. 10.1177/1609406920969268

[36] Genuis SJ. An ounce of prevention: a pound of cure for an ailing health care system. Can Fam Physician. (2007) 53:597–99. Available online at: https://www.cfp.ca/content/53/4/597.short (accessed March 30, 2022). 17872698 PMC1952577

[37] LaDonna KA Taylor T Lingard L. Why open-ended survey questions are unlikely to support rigorous qualitative insights. Acad Med. (2018) 93:347–9. 10.1097/ACM.0000000000002088 29215376

[38] Lindgren B-M Lundman B Graneheim UH. Abstraction and interpretation during the qualitative content analysis process. Int J Nurs Stud. (2020) 108:103632. 10.1016/j.ijnurstu.2020.103632 32505813

[39] WSAVA. World Small Animal Veterinary Association Global Dental Guidelines. (2018). Available online at: https://onlinelibrary.wiley.com/doi/epdf/10.1111/jsap.13132.

[40] Fons-Badal C Fons-Font A Labaig-Rueda C Fernanda Solá-Ruiz M Selva-Otaolaurruchi E Agustín-Panadero R. Analysis of predisposing factors for rapid dental calculus formation. J Clin Med. (2020) 9:858. 10.3390/jcm9030858 32245069 PMC7141533

[41] Rawlings JM Culham N. Halitosis in dogs and the effect of periodontal therapy. J Nutr. (1998) 128:2715S−6S. 10.1093/jn/128.12.2715S 9868249

[42] Buckley C Colyer A Skrzywanek M Jodkowska K Kurski G Gawor J. The impact of home-prepared diets and home oral hygiene on oral health in cats and dogs. Br J Nutr. (2011) 106:S124–S7. 10.1017/S0007114511000821 22005407

[43] VOHC VOHC. VOHC_Accepted_Products (2021). Available online at: http://www.vohc.org/.

[44] Freeman LM Chandler ML Hamper BA Weeth LP. Current knowledge about the risks and benefits of raw meat–based diets for dogs and cats. J Am Vet Med Assoc. (2013) 243:1549–58. 10.2460/javma.243.11.1549 24261804

[45] Morelli G Bastianello S Catellani P Ricci R. Raw meat-based diets for dogs: survey of owners' motivations, attitudes and practices. BMC Vet Res. (2019) 15:1–10. 10.1186/s12917-019-1824-x 30832667 PMC6399943

[46] Hielm-Bjorkman A Virtanen J. Exploratory study: 632 shared experiences from dog owners changing their dogs' food to a raw food (BARF) diet. Link (2019). Available online at: https://www2.helsinki.fi/sites/default/files/atoms/files/kokemuksia_raakaruokinnasta.pdf (accessed March 31, 2022).

[47] Robinson J Gorrel C editors. The oral status of a pack of foxhounds fed a “natural” diet. In: Proceedings of the Fifth World Veterinary Dental Congress, Birmingham (1997). p. 35–37.

[48] Axelsson E Ratnakumar A Arendt M-L Maqbool K Webster MT Perloski M. The genomic signature of dog domestication reveals adaptation to a starch-rich diet. Nature. (2013) 495:360–4. 10.1038/nature11837 23354050

[49] Soukup JW Hetzel S Paul A. Classification and epidemiology of traumatic dentoalveolar injuries in dogs and cats: 959 injuries in 660 patient visits (2004–2012). J Vet Dent. (2015) 32:6–14. 10.1177/089875641503200101 26197685

[50] Soltero-Rivera M Elliott MI Hast MW Shetye SS Castejon-Gonzalez AC Villamizar-Martinez LA. Fracture limits of maxillary fourth premolar teeth in domestic dogs under applied forces. Front Vet Sci. (2019) 5:339. 10.3389/fvets.2018.00339 30761310 PMC6364561

[51] Pinto CFD Lehr W Pignone VN Chain CP Trevizan L. Evaluation of teeth injuries in Beagle dogs caused by autoclaved beef bones used as a chewing item to remove dental calculus. PLoS ONE. (2020) 15:e0228146. 10.1371/journal.pone.0228146 32053619 PMC7018081

[52] van Veggel N Armstrong M. In dogs with periodontal disease is feeding a complete raw meat diet more effective than a complete Kibble'dental'diet at reducing periodontal disease? Vet Evid. (2017) 2. 10.18849/ve.v2i2.88

[53] Lindhe J Wicén PO. The effects on the gingivae of chewing fibrous foods. J Periodontal Res. (1969) 4:193–201. 10.1111/j.1600-0765.1969.tb01966.x 4244252

[54] Rubido S García-Caballero L Abeleira MT Limeres J García M Diz P. Effect of chewing an apple on dental plaque removal and on salivary bacterial viability. PLoS ONE. (2018) 13:e0199812. 10.1371/journal.pone.0199812 30020943 PMC6051571

[55] Janssens L Verhaert L Berkowic D Adriaens D. A standardized framework for examination of oral lesions in wolf skulls (Carnivora: Canidae: Canis lupus). J Mammal. (2016) 97:1111–24. 10.1093/jmammal/gyw058

[56] Pires AE Caldeira IS Petrucci-Fonseca F Viegas I Viegas C Bastos-Silveira C. Dental pathology of the wild Iberian wolf (Canis lupus signatus): The study of a 20th century Portuguese museum collection. Vet Anim Sci. (2020) 9:100100. 10.1016/j.vas.2020.100100 32734110 PMC7386764

[57] Steenkamp G Gorrel C. Oral and dental conditions in adult African wild dog skulls: a preliminary report. J Vet Dent. (1999) 16:65–8. 10.1177/089875649901600201 10863513

[58] Olsén L Brissman A Wiman S Eriksson F Kaj C Brunius Enlund K. Improved oral health and adaptation to treatment in dogs using manual or ultrasonic toothbrush or textile of nylon or microfiber for active dental home care. Animals. (2021) 11:2481. 10.3390/ani11092481 34573447 PMC8469497

[59] Michie S Van Stralen MM West R. The behaviour change wheel: a new method for characterising and designing behaviour change interventions. Implement Sci. (2011) 6:1–12. 10.1186/1748-5908-6-42 21513547 PMC3096582

